# What to report in sellar tumor MRI? A nationwide survey among German pituitary surgeons, radiation oncologists, and endocrinologists

**DOI:** 10.1007/s00234-023-03222-w

**Published:** 2023-09-22

**Authors:** Torge Huckhagel, Christian Riedel, Jörg Flitsch, Roman Rotermund

**Affiliations:** 1 Department of Diagnostic and Interventional Neuroradiology, University Medical Center Göttingen, Göttingen, Germany; 2 Department of Neurosurgery, Division of Pituitary Surgery, University Medical Center Hamburg-Eppendorf, Hamburg, Germany; 3 Department of Neurosurgery, Diako Krankenhaus Flensburg, Flensburg, Germany

**Keywords:** Brain neoplasms, Craniopharyngioma, Magnetic resonance imaging, Meningioma, Pituitary neoplasms, Sella turcica

## Abstract

**Purpose:**

While MRI has become the imaging modality of choice in the diagnosis of sellar tumors, no systematic attempt has yet been made to align radiological reporting of findings with the information needed by the various medical disciplines dealing with these patients. Therefore, we aimed to determine the prevailing preferences in this regard through a nationwide expert survey.

**Methods:**

First, an interdisciplinary literature-based catalog of potential reporting elements for sellar tumor MRI examinations was created. Subsequently, a web-based survey regarding the clinical relevance of these items was conducted among board certified members of the German Society of Neurosurgery, German Society of Radiation Oncology, and the Pituitary Working Group of the German Society of Endocrinology.

**Results:**

A total of 95 experts (40 neurosurgeons, 28 radiation oncologists, and 27 endocrinologists) completed the survey. The description of the exact tumor location, size, and involvement of the anatomic structures adjacent to the sella turcica (optic chiasm, cavernous sinus, and skull base), occlusive hydrocephalus, relationship to the pituitary gland and infundibulum, and certain structural characteristics of the mass (cyst formation, hemorrhage, and necrosis) was rated most important (> 75% agreement). In contrast, the characterization of anatomic features of the nasal cavity and sphenoid sinus as well as the findings of advanced MRI techniques (e.g., perfusion and diffusion imaging) was considered relevant by less than 50% of respondents.

**Conclusion:**

To optimally address the information needs of the interdisciplinary treatment team, MRI reports of sellar masses should primarily focus on the accurate description of tumor location, size, internal structure, and involvement of adjacent anatomic compartments.

**Supplementary Information:**

The online version contains supplementary material available at 10.1007/s00234-023-03222-w.

## Introduction

Pituitary fossa and craniopharyngeal duct tumors account for 16.6% of all primary brain and central nervous system tumors, according to the Central Brain Tumor Registry of the United States (CBTRUS) statistical report 2008–2012 [1]. Most of these pathologies are histologically benign, with pituitary adenomas representing the most common entities [1, 2]. While computed tomography is still useful in assessing the osseous anatomical conditions of the skull base and tumor calcifications, it has been largely replaced by magnetic resonance imaging (MRI) as the imaging modality of choice in the evaluation of sellar masses due to its higher soft tissue contrast [3, 4]. With steady development of imaging techniques and equipment including high-field 3-T MRI, anatomical relationships and pathologies can be described with increasing detail. Based on these prerequisites, experts reported recommendations both for the execution of MRI examinations of hypophyseal fossa tumors and for the content of respective radiology reports [4–7]. To the best of our knowledge, however, no attempt has been made so far to correlate the content of radiology reports with the information needed by the interdisciplinary team dealing with sellar tumor patients, although such a single-center initiative has already resulted in increased satisfaction among referring physicians in the case of primary brain tumors at Emory University School of Medicine, Atlanta, Georgia, USA [8, 9]. Therefore, we aimed to determine the specific content preferences for MRI reports of neoplastic lesions of the sellar region by board certified neurosurgeons, radiation oncologists, and endocrinologists in a nationwide survey.

## Methods

### Ethics and study design

The institutional ethics committee reviewed the study protocol, and ethics approval was waived (registration number 9/2/21), since it is not a medical research project on humans and therefore does not formally require approval. In terms of study design, this is a nationwide structured online poll among German board certified specialists in the fields of neurosurgery, radiation oncology, and endocrinology who are regularly involved in the diagnosis and treatment of patients with space-occupying lesions of the sellar region. Data were collected prospectively in the form of a cross-sectional survey. Where appropriate and possible, the design of the study and presentation of the results follow the recommendations for good practice in the conduct and reporting of survey research as outlined by Kelley and colleagues [10].

### Course of study

**Fig. 1:**
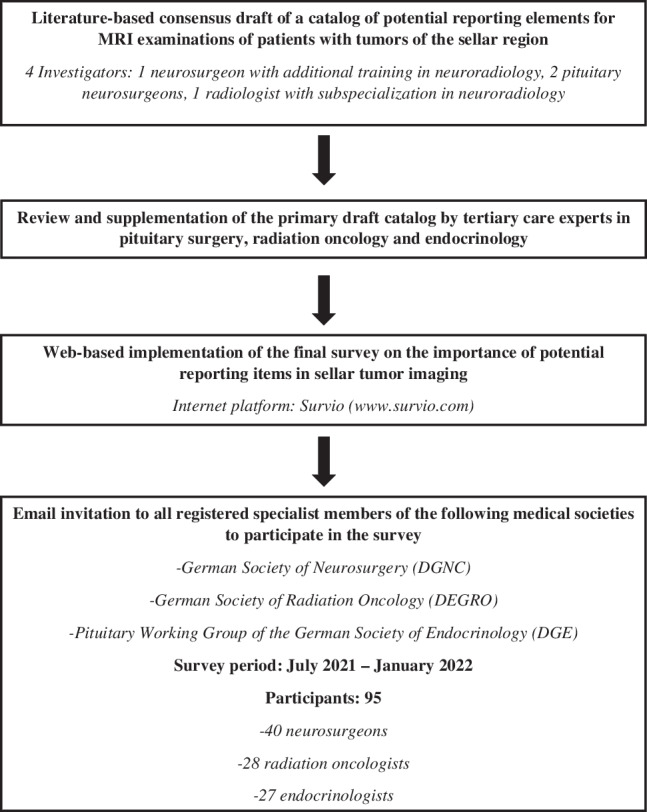
Synopsis of the study procedures

First, based on a comprehensive search of the international English- and German-language neuroradiological and neurosurgical literature, a comprehensive list of potential reporting elements for MRI examinations of patients with tumors of the pituitary fossa and/or adjacent anatomical regions was compiled by the principal investigator (TH), being a board certified neurosurgeon with additional two years of training in clinical neuroradiology. To achieve generality in this regard, aspects of both pediatric and adult tumors were included. Subsequently, this catalog was expanded to include experiences of all co-authors, senior neurosurgeons with long-standing pituitary surgery experience (JF, RR), and a senior radiologist with sub-specialization in neuroradiology (CR). In a following pilot phase, specialist colleagues from the various disciplines involved evaluated the individual categories in terms of their comprehensibility and completeness. After incorporating their feedback suggestions, we implemented the final questionnaire on the online platform Survio (www.survio.com). The structure of the questionnaire was designed in such a way that it asks the respondent to classify each potential MRI reporting item as either “essential” or “non-essential” with respect to their clinical practice experience. A sample questionnaire is available as Supplement 1 to this publication. Thanks to the active support of the German Society of Neurosurgery (DGNC), German Society of Radiation Oncology (DEGRO), and the Pituitary Working Group of the German Society of Endocrinology (DGE), all registered board certified specialist members could be invited to participate in the online survey via email between July 2021 and January 2022. Potential candidates were asked to participate in the poll only if they had practical experience in the diagnosis and/or treatment of patients with tumors of the sellar region. A brief synopsis of the study course is presented in Fig. 1 (Synopsis of the study procedures).

### Study participants

A total of 95 medical specialists completed the online questionnaire. All responses were submitted between 7/24/2021 and 1/25/2022 (186 days). Most participants (83.5%) required 10 min or less to complete. Broken down by discipline, 40 (42.1%) pituitary neurosurgeons, 28 (29.5%) radiation oncologists, and 27 (28.4%) endocrinologists participated in the study.

### Data presentation and statistics

Cumulative and discipline-specific survey results are presented descriptively as numerical ratios (number of positive ratings/number of respondents) and percentages, respectively. For selected clinically meaningful findings, the associated 95% confidence intervals (CI) are additionally included as measures of dispersion (modified Wald method). The assessments of the separate medical disciplines (neurosurgery, radiation oncology, and endocrinology) were compared for each potential MRI reporting item using a two-sided Chi-square test. A *p* value < 0.05 was considered statistically significant. All statistical procedures were performed using GraphPad Prism version 9.4.1 for Windows (GraphPad Software, San Diego, California, USA; www.graphpad.com).

## Results

### Cumulative assessment of MRI reporting items

Among the most significant reporting elements (rated as essential by > 75% of all participating experts) are primarily those related to the exact tumor location, internal structure, size/expansion, and the impact of the mass on surrounding anatomic structures. When measuring tumor size, respondents largely preferred to consider all areas, including cystic and necrotic parts, as opposed to reporting only the contrast enhancing pathologic tissue (87/95; 91.6%; CI 84.0–95.9%). Besides compartmental tumor extension (93/95; 97.9%; CI 92.2–99.9%), specifically optic chiasm impairment (95/95; 100.0%; CI 95.3–100.0%), cavernous sinus infiltration (93/95; 97.9%; CI 92.2–99.9%), and occlusive hydrocephalus (92/95; 96.8%; CI 90.7–99.3%) showed the highest agreement scores. Other specific elements regarding adjacent structures, which were classified by at least 3 of 4 respondents as essential to an MRI report, comprise invasion/erosion of the skull base (91/95; 95.8%; CI 89.3–98.7%) and anatomic tumor-pituitary gland relationship (86/95; 90.5%; CI 82.8–95.1%), including deviation of the infundibulum (74/95; 77.9%; CI 68.5–85.1%). Important characteristics concerning the internal structure of the mass reaching > 75% overall agreement are cyst formation (88/95; 92.6%; CI 85.3–96.6%), intralesional hemorrhage (87/95; 91.6%; CI 84.0–95.9%), and necrosis (77/95; 81.1%; CI 71.9–87.8%).

Other tumor features on MRI, which had a moderate overall support of 50–75%, relate on the one hand to structural characteristics of the lesion such as delineation of tumor margin, tumor signal properties (homogeneity) in the T2 weighted imaging, calcifications, and contrast enhancement pattern and, on the other hand, further effect of the mass on surrounding anatomic structures. These include potential elevation of the sellar diaphragm, reactive thickening of the adjacent dura mater (dural tail), bone remodeling, more detailed considerations regarding cavernous sinus infiltration in the sense of the application of the Knosp classification (likelihood of cavernous sinus invasion), and the description of a possible tumor-associated stenosis of the cavernous internal carotid artery.

Of lower value (< 50% agreement) to the experts interviewed were the integration of information on tumor internal structure in nonenhanced T1 weighted images, the use of Hardy classification (sellar enlargement/invasion), advanced imaging techniques (tumor perfusion/diffusion features, diffusion tensor imaging of the optic nerve/chiasm), and anatomical particularities of the nasal cavity or sphenoid sinus.

**Fig. 2:**
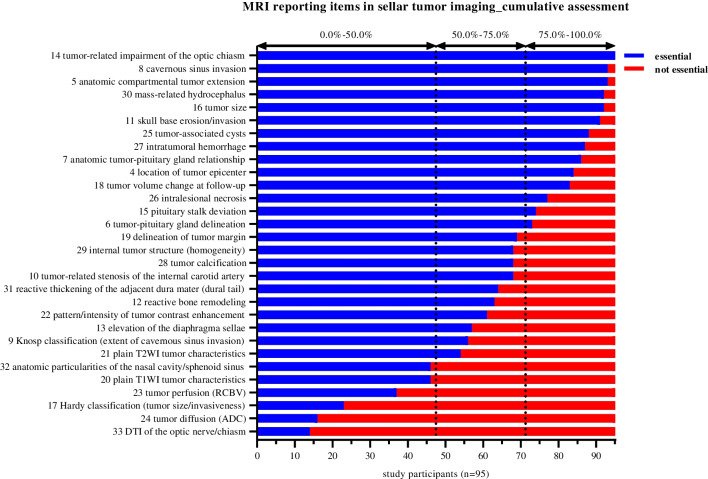
Cumulative assessment of potential MRI reporting items in patients with sellar tumors

More in-depth information about the assessment of all investigated imaging elements can be obtained from Fig. 2 (cumulative assessment of potential MRI reporting items in patients with sellar tumors) and Table 1 (discipline-specific rating of potential MRI reporting elements in patients with sellar tumors).

**Table 1:** Discipline-specific rating of potential MRI reporting elements in patients with sellar tumors

MRI reporting items	Neurosurgeons (*n* = 40)	Radiation oncologists (*n* = 28)	Endocrinologists (*n* = 27)	All (*n* = 95)	*p* value
4 location of tumor epicenter	31/40 (77.5%)	27/28 (96.4%)	26/27 (96.3%)	84/95 (88.4%)	**0.0179**
5 anatomic compartmental tumor extension	39/40 (97.5%)	28/28 (100.0%)	26/27 (96.3%)	93/95 (97.9%)	0.6166
6 tumor-pituitary gland delineation	28/40 (70.0%)	21/28 (75.0%)	24/27 (88.9%)	73/95 (76.8%)	0.1913
7 anatomic tumor-pituitary gland relationship	37/40 (92.5%)	25/28 (89.3%)	24/27 (88.9%)	86/95 (90.5%)	0.8537
8 cavernous sinus invasion	40/40 (100.0%)	26/28 (92.9%)	27/27 (100.0%)	93/95 (97.9%)	0.0868
9 Knosp classification (extent of cavernous sinus invasion)	28/40 (70.0%)	13/28 (46.4%)	15/27 (55.6%)	56/95 (58.9%)	0.1380
10 tumor-related stenosis of the internal carotid artery	26/40 (65.0%)	19/28 (67.9%)	23/27 (85.2%)	68/95 (71.6%)	0.1739
11 skull base erosion/invasion	38/40 (95.0%)	28/28 (100.0%)	25/27 (92.6%)	91/95 (95.8%)	0.3722
12 reactive bone remodeling	28/40 (70.0%)	17/28 (60.7%)	18/27 (66.7%)	63/95 (66.3%)	0.7269
13 elevation of the diaphragma sellae	27/40 (67.5%)	7/28 (25.0%)	23/27 (85.2%)	57/95 (60.0%)	**0.0001**
14 tumor-related impairment of the optic chiasm	40/40 (100.0%)	28/28 (100.00%)	27/27 (100.0%)	95/95 (100.0%)	NA
15 pituitary stalk deviation	33/40 (82.5%)	15/28 (53.6%)	26/27 (96.3%)	74/95 (77.9%)	**0.0004**
16 tumor size	38/40 (95.0%)	27/28 (96.4%)	27/27 (100.0%)	92/95 (96.8%)	0.5117
17 Hardy classification (tumor size/invasiveness)	9/40 (22.5%)	7/28 (25.0%)	7/27 (25.9%)	23/95 (24.2%)	0.9434
18 tumor volume change at follow-up	33/40 (82.5%)	24/28 (85.7%)	26/27 (96.3%)	83/95 (87.4%)	0.2371
19 delineation of tumor margin	26/40 (65.0%)	22/28 (78.6%)	21/27 (77.8%)	69/95 (72.6%)	0.3626
20 plain T1WI tumor characteristics	16/40 (40.0%)	17/28 (60.7%)	13/27 (48.1%)	46/95 (48.4%)	0.2428
21 plain T2WI tumor characteristics	25/40 (62.5%)	15/28 (53.6%)	14/27 (51.9%)	54/95 (56.8%)	0.6319
22 pattern/intensity of tumor contrast enhancement	30/40 (75.0%)	12/28 (42.9%)	19/27 (70.4%)	61/95 (64.2%)	**0.0181**
23 tumor perfusion (RCBV)	10/40 (25.0%)	10/28 (35.7%)	17/27 (63.0%)	37/95 (38.9%)	**0.0069**
24 tumor diffusion (ADC)	6/40 (15.0%)	6/28 (21.4%)	4/27 (14.8%)	16/95 (16.8%)	0.7420
25 tumor-associated cysts	39/40 (97.5%)	22/28 (78.6%)	27/27 (100.0%)	88/95 (92.6%)	**0.0030**
26 intralesional necrosis	32/40 (80.0%)	19/28 (67.9%)	26/27 (96.3%)	77/95 (81.1%)	**0.0261**
27 intratumoral hemorrhage	40/40 (100.0%)	21/28 (75.0%)	26/27 (96.3%)	87/95 (91.6%)	**0.0007**
28 tumor calcification	32/40 (80.0%)	14/28 (50.0%)	22/27 (81.5%)	68/95 (71.6%)	**0.0105**
29 internal tumor structure (homogeneity)	29/40 (72.5%)	16/28 (57.1%)	23/27 (85.2%)	68/95 (71.6%)	0.0692
30 mass-related hydrocephalus	39/40 (97.5%)	27/28 (96.4%)	26/27 (96.3%)	92/95 (96.8%)	0.9519
31 reactive thickening of the adjacent dura mater (dural tail)	32/40 (80.0%)	23/28 (82.1%)	9/27 (33.3%)	64/95 (67.4%)	** < 0.0001**
32 anatomic particularities of the nasal cavity/sphenoid sinus	27/40 (67.5%)	11/28 (39.3%)	8/27 (29.6%)	46/95 (48.4%)	**0.0050**
33 DTI of the optic nerve/chiasm	4/40 (10.0%)	7/28 (25.0%)	3/27 (11.1%)	14/95 (14.7%)	0.1879

40 neurosurgeons, 28 radiation oncologists, and 27 endocrinologists participated in the survey. The fractions/percentages reflect the participants’ ranking of each item as essential to the MRI report. The numbers preceding the MRI reporting items represent the numbers of the associated questions in the underlying online survey. Statistically significant group differences (*p* < 0.05; two-sided Chi-square test) are written in bold type. *ADC*, apparent diffusion coefficient; *DTI*, diffusion tensor imaging; *NA*, not available; *RCBV*, relative cerebral blood volume; *T1WI*, T1 weighted imaging; *T2WI*, T2 weighted imaging

**Fig. 3:**
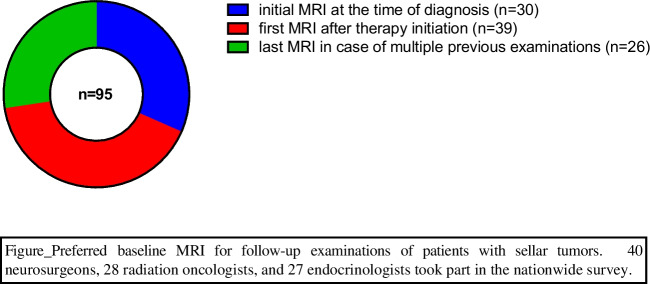
Preferred baseline MRI for follow-up examinations of patients with sellar tumors

A mixed opinion with no clear trend emerged when asked about the preferred baseline MRI scan to be used for comparison during follow-up imaging, as shown in Fig. 3 (preferred baseline MRI for follow-up examinations of patients with sellar tumors).

### Interdisciplinary differences in prioritization of potential MRI reporting items

**Fig. 4:**
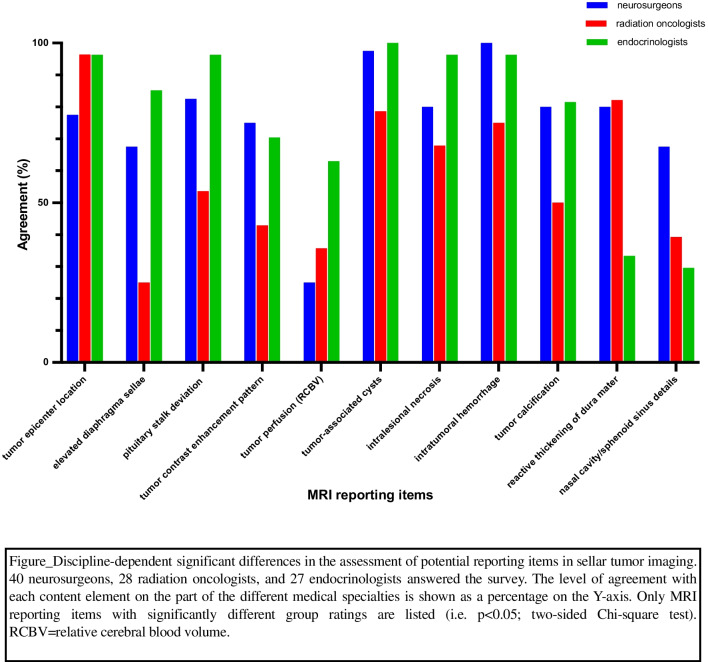
Discipline-dependent significant differences in the assessment of potential reporting items in sellar tumor imaging

Pituitary surgeons and endocrinologists together, compared with radiation oncologists, were by and large more interested in characteristics of the tumor internal composition, including gadolinium uptake behavior and the presence of lesion-associated cysts, calcifications, necrosis, and hemorrhage (all *p* < 0.05). Interestingly, in contrast to the other two groups, the majority of radiotherapists rated the contrast agent uptake pattern/intensity as negligible (approval rate 12/28; 42.9%; CI 26.5–61.0%), whereas for the other abovementioned parameters, a significant difference was found only with regard to the extent of consent with an overall majority agreement in all groups (> / = 50.0%). While elevation of the diaphragma sellae was of primary importance only to endocrinologists (23/27; 85.2%; CI 66.9–94.7%) and neurosurgeons (27/40; 67.5%; CI 51.9–80.0%), reactive thickening of the adjacent dura mater was considered essential solely by radiation oncologists (23/28; 82.1%; CI 63.9–92.6%) and neurosurgeons (32/40; 80.0%; CI 65.0–89.8%). Tumor perfusion was categorized as relevant to the MRI report merely by the participating endocrinologists (17/27; 63.0%; CI 44.2–78.5%) and anatomical particularities of the nasal cavity or sphenoid sinus only by the interviewed pituitary surgeons (27/40; 67.5%; CI 51.9–80.0%). All potential MRI reporting items with significantly divergent ratings (i.e., *p* < 0.05) by the different involved medical specialties are shown in Fig. 4 (discipline-dependent significant differences in the assessment of potential reporting items in sellar tumor imaging) for a better overview.

## Discussion

Through the extensive survey presented in this study, we explore for the first time on a national scale the demands that treating clinicians place on MRI reports in the context of caring for patients with tumors of the sellar region. Although several recommendations have already been published in the past by experienced neuroradiologists on the realization and reporting of MRI scans for the evaluation of sellar masses [6, 7, 11–13], only very few of them are based on a consensus or on a systematic survey and analysis of the information needs and expectations of the treating referring specialists who are the primary target audience for the MRI reports [14, 15]. In fact, it appears that to date the desire for standardized style and billing considerations influence the development of structured radiology reporting to a far greater extent than do the requirements of referrers, as a national survey of academic radiologists in the United States of America showed [16]. While this article primarily focuses on the provision of high quality radiology reports that meet the information needs of clinicians, it should not be ignored that this can only be achieved by the radiologist through a sufficient information base in the form of an adequate patient history and justification for imaging by the referring clinician [17]. Whereas in modern market economies, the wishes of consumers or customers play a central role in the production of a commodity or the provision of a service; few attempts have been made by radiological protagonists to determine the satisfaction of referring physicians as customers of the product “MRI report” or to optimize the radiological report regarding existing needs [16]. Only a small number of studies have so far taken stock of the situation and revealed deficits in the reporting from the point of view of the addressees. For example, a survey of general practitioners in the United Kingdom revealed that the majority were not familiar with the normal size ranges of frequently measured and reported anatomical structures, and a survey of medical oncologists in Australia revealed that key lesion sizes, which they considered very important, were often not available and that a desired comparison with previous examinations was often not carried out [18, 19]. Boll and colleagues took a structured approach to this problem and, using the so-called “voice-of-the-customer method,” were able to show at their institution that the greatest deficits perceived by clinicians in radiology reports were insufficient consideration of their specific information needs and the lack of communication of key information relevant to practice [20]. This feedback should be considered by clinical radiologists as a valuable tool with a potential for qualitative and quantitative enhancement of their reporting activities. The basic idea of a systematic orientation toward the specific information needs of referring clinicians has so far been taken up in the neuroradiological-oncological context only for primary brain tumors (gliomas), on the one hand, monocentrically at Emory University (Atlanta/USA) and, on the other hand, in the context of a nationwide survey among neurosurgeons, radiation therapists, medical oncologists, and neuropathologists in Germany [9, 21]. Implementation of the so-called “brain tumor reporting and data system (BT-RADS)” resulted in higher satisfaction among clinicians with radiologic image analysis through improved coherence, unambiguity, and interdisciplinary communication in a follow-up of the first-mentioned study after the MRI reporting elements were adapted to the preferences of referring physicians [8]. In addition, it has already been shown in other subdisciplines that radiologists also prefer department-wide standardized structured reporting after its pervasive implementation in the longer term. According to Larson et al., this could only be achieved by closely involving all medical staff of the radiology department in the initial template creation process, avoidance of excessive restriction of reporting by a certain degree of flexibility in describing abnormal findings, and continuously considering user feedback after the introduction of the templates, since there was initially a certain skepticism due to the anticipated loss of autonomy [22]. Following this guiding principle, we present here an evaluation of potential MRI reporting elements for patients with tumors of the sellar region, which could be collected for the first time through a nationwide survey among clinical experts in the disciplines of neurosurgery, radiotherapy, and endocrinology relevant to the management of these conditions [23]. Our survey results are largely consistent with the consensus-based proposal of a small ENT- and skull base surgery-oriented expert group (3 neuroradiologists, 3 ENT specialists, and 3 skull base surgeons) from Melbourne for a structured pituitary MRI reporting template, which also includes adenoma size, internal composition of the lesion, relationship to pituitary tissue/infundibulum, and impairment of adjacent structures (optic chiasm, cavernous sinus, and internal carotid artery) [14]. This template lists detailed characterization of the sphenoid sinus (size and pneumatization) and the nasal cavity (septal deviation, Onodi cells, and changes due to previous sinonasal surgery) as essential additional reporting elements. Although these items were rated as important in the overall evaluation in our study by only a minority, they received significant agreement among the neurosurgeons surveyed. This is understandable given that transsphenoidal surgery is currently the standard surgical approach for the vast majority of sellar tumors, and anatomic abnormalities of the sphenoid sinus have the potential for serious intraoperative complications (e.g., injury to the internal carotid artery) [23–26]. In this context, it should also be mentioned that a joint project of the American Society of Neuroradiology with the American College of Radiology and the Radiological Society of North America has created a set of common data elements for pituitary microadenomas, which similarly focuses on fundamental aspects that were identified as essential in our expert survey (including tumor location, size, contrast enhancement, and infundibulum abnormalities) [27, 28]. According to the experts interviewed, the tumor signal characteristics in the non-contrast T2 weighted imaging as well as T1 sequence after gadolinium application are significantly more relevant compared to the plain T1 weighted image. However, regarding the value of the different MRI sequences, the pertinent literature is quite heterogeneous. While Kumar et al. emphasize the importance of the non-enhanced T1 sequence and Karimian-Jazi the relevance of the dynamic T1 weighted imaging after contrast agent application for the detection of microadenomas, Bonneville considers the plain T2 sequence often more informative in pituitary imaging [6, 12, 29]. For tumor sizing, the clinical experts voted for the inclusion not only of the contrast-enhancing lesions, but additionally of any tumor cysts and necrotic areas present. This contrasts with the general practice for malignant gliomas, where, according to RANO criteria (Response Assessment in Neuro-Oncology), tumor cysts are generally not included in the measurement [30]. It should be noted here, though, that despite expansion of RANO efforts to include numerous neuro-oncology fields of work—such as low-grade gliomas, brain metastases, leptomeningeal metastases, meningiomas, and spinal neoplasms—no such recommendations have yet been developed for pituitary tumors [31]. Even beyond the RANO working groups, no standardized radiological criteria for the estimation of treatment response for pituitary tumors are available to date. However, in a recent study, Imber and colleagues were able to demonstrate adequate correlation of one- and two-dimensional measurement techniques with the volumetric gold standard, even for irregularly configured adenomas [32]. The utilization of the Hardy classification for a more detailed characterization of pituitary adenomas was rejected by most respondents. Originally developed in the 1970s using conventional radiographic techniques, significant limitations in terms of its reliability have been noted in the era of MRI despite frequent use in studies, so skepticism seems justified [33, 34]. Moreover, with a precise description of the tumor extension into the adjacent intracranial compartments and the skull base, all rated as essential by the participants, the key information contained in the Hardy classification is conveyed, so that a separate mention seems in principle dispensable. Thickening of the dura mater adjacent to the tumor (so-called dural tail) was considered an important element by neurosurgeons and radiotherapists in contrast to endocrinologists. This becomes understandable by the fact that besides an inflammatory origin, tumor cell infiltrates may well be present, which may have implications for local treatment modalities [35–37]. Advanced imaging modalities such as MR perfusion and diffusion were also not considered integral parts of MRI protocols of sellar region tumors by the majority of participating experts. This is basically in line with contemporary recommendations from the related literature, in which performing thin-slice anatomic sequences (T1 weighted imaging with and without contrast and T2 weighted imaging) in multiple planes is considered a diagnostic imaging standard [3, 6, 7]. The Congress of Neurological Surgeons recommendations on imaging for nonfunctional pituitary adenomas emphasize the currently unclear importance of diffusion imaging regarding its correlation with tumor firmness, but also cite low-evidence findings that various MR perfusion techniques may provide information about adenoma vascularization, which could be valuable in terms of surgical planning and predicting the risk of postoperative bleeding [38]. Respondents had no clear preference on the question of the baseline to be selected for follow-up MRI. While imaging prior to initiation of therapy was chosen as a reference in a study on the response of invasive prolactinomas to bromocriptine, the current guideline of the AWMF (Association of the Scientific Medical Societies in Germany) advocates assessing tumor progression in comparison with the immediate past examination. Another option would be to choose the nadir of tumor extension as a comparator, as suggested by the RANO clinical trials working group in meningioma patients [39–41].

### Strengths and limitations

The main value of the study is that here, for the first time, the clinical requirements for a radiological report in the context of sellar tumor imaging could be identified with the help of a nationwide interdisciplinary survey among experts from the fields of pituitary surgery, radiotherapy, and endocrinology. In contrast, previous recommendations have been published either by individual experienced neuroradiological authors or, at best, by small groups of experts [14]. In the present work, we have focused primarily on the experience of the specialists and not on the institutional affiliation (academic versus non-academic), although a further breakdown might have yielded additional interesting insights. Nevertheless, a recent study shows that surgical treatment of pituitary tumors in Europe is mainly performed at academic centers and here again predominantly by only a few highly specialized and trained pituitary surgeons [42]. In this respect, it may be assumed that most of the study participants are related to academic centers, since regular extensive involvement in the treatment of patients with sellar tumors was a prerequisite for the experts to participate in the survey. In principle, the design of a preconfigured questionnaire with a defined set of items raises the possibility of incomplete thematic coverage. We attempted to prevent this potential bias by giving all participants the opportunity at the end of the survey to submit their own supplementary requests in free-text format. However, all the suggestions made were merely individual opinions without recurring aspects, which means that integration into a general reporting standard does not seem warranted. On the other hand, the provision of extensive selection options could also have led to a skewed increase in information request among survey respondents according to the well-known principle that a given supply creates its demand. The primary intention of the study is to provide a recommendation as to what should be included in the MRI report according to the “voice-of-the-customer method” rather than suggest what should be left out to ensure that all contextual clinical information needs are met. In this context, it must be pointed out that additional image information, which may not be considered essential by the referring physicians, may still be important for the interpreting radiologist to finally make the correct differential diagnosis. The present work is explicitly limited to MRI as the current gold standard in the imaging of sellar lesions. For this reason, no direct conclusions should be drawn from it with regard to other potentially relevant diagnostic modalities, such as computed tomography or positron emission tomography. Furthermore, we spatially restricted the project to the (para)sellar region, so that certain tumor entities, such as multifocal germinomas which in addition to their perisellar manifestation are often also found adjacent to the pineal gland or show leptomeningeal dissemination, are not completely covered by the provided focused reporting categories with regard to the latter manifestations [7].

## Conclusions

The radiological report is a central communication tool in the interdisciplinary management of patients with space-occupying lesions of the sellar region. To meet the information needs of the involved clinical disciplines, MRI reporting should focus primarily on describing the location of the tumor epicenter, the size and exact compartmental extension, the associated impact on neighboring anatomical structures (especially the normal pituitary tissue/infundibulum, optic chiasm, cavernous sinus, internal carotid artery, and adjacent skull base), the internal characteristics of the mass, and the exclusion of complicating obstructive hydrocephalus. Apart from these basic requirements demanded by clinicians, additional characteristics may be relevant to the radiologist in making the correct differential diagnosis. Consistent adherence to a demand-oriented reporting checklist has the potential to improve interdisciplinary information exchange and thereby make a positive contribution to the care of this patient population.

### Supplementary Information

Below is the link to the electronic supplementary material.

Supplementary file1 (PDF 81 KB)

## Data Availability

All relevant information collected in this study is included in the manuscript or in the tables and figures. Metadata are available from the corresponding author upon reasonable request.

## Abbreviations

*CI*: 95% Confidence interval
*MRI*: Magnetic resonance imaging
