# Supplementary material for: What to report in sellar tumor MRI? A nationwide survey among German pituitary surgeons, radiation oncologists, and endocrinologists
Source: Neuroradiology. 2023 Sep 22;65(11):1579–88. doi: 10.1007/s00234-023-03222-w (PMC10567906; doi:10.1007/s00234-023-03222-w)
Supplement: Supplementary file 1 — Supplementary file1 (PDF 81 KB) [file 234_2023_3222_MOESM1_ESM.pdf]

Anhang: Umfrage

MRT-Befunde von Tumoren der Sellaregion

Sehr geehrte Kolleginnen und Kollegen,

bitte nehmen Sie sich ein paar Minuten Zeit, um die folgende Umfrage der Universitätsmedizin Göttingen zu MRT-Befunden von Tumoren der Sellaregion auszufüllen. Herzlichen Dank für ihre Unterstützung!

1 Bitte bestätigen sie vor Beginn der Umfrage ihr Einverständnis zur freiwilligen Teilnahme. Die gewonnenen Informationen werden vertraulich und anonymisiert für wissenschaftliche Zwecke ausgewertet. Das vorliegende Projekt wurde von der Ethik-Kommission der Universitätsmedizin Göttingen begutachtet.

Fragesupport: *Wählen sie bitte eine Antwort aus*

◯ Ich bestätige meine freiwillige Teilnahme ◯ Ich möchte an der Umfrage nicht teilnehmen

2 Diese Umfrage richtet sich an fachärztliche Kollegen aus den Bereichen Endokrinologie, Strahlentherapie und Neurochirurgie. Bitte geben sie an, in welchem Fachbereich sie tätig sind.

Fragesupport: *Wählen sie bitte eine Antwort aus*

☐ Endokrinologie ☐ Strahlentherapie ☐ Neurochirurgie

3 Sind sie im Rahmen ihrer klinischen Tätigkeit an der Diagnostik oder Behandlung von Patienten mit Tumoren der Sellaregion (z.B. Hypophysenadenome, Kraniopharyngeome) beteiligt?

Fragesupport: *Wählen Sie bitte eine Antwort aus*

◯ Ja ◯ Nein (Beendigung des Fragebogens)

4 Die Beschreibung der Lokalisation der Haupttumormasse (sogenanntes Epizentrum) ist für mich

Fragesupport: *Wählen sie bitte eine Antwort aus*

◯ wesentlicher Bestandteil des MRT-Befundes ◯ kein wesentlicher Bestandteil des MRT-Befundes

5 Die genaue anatomische Ausdehnung der Raumforderung (intra-, supra-, infra- und parasellär) ist für mich ...

Fragesupport: *Bitte wählen sie eine Antwort aus*

◯ wesentlicher Bestandteil des MRT-Befundes ◯ kein wesentlicher Bestandteil des MRT-Befundes

6 Die Erwähnung der Abgrenzbarkeit von Adeno- und Neurohypophyse gegenüber dem Tumor ist für mich ...

Fragesupport: *Bitte wählen sie eine Antwort aus*

◯ wesentlicher Bestandteil des MRT-Befundes ◯ kein wesentlicher Bestandteil des MRT-Befundes

7 Die Beschreibung der anatomischen Lagebeziehung der Raumforderung zum Hypophysengewebe ist für mich ...

Fragesupport: *Bitte wählen sie eine Antwort aus*

◯ wesentlicher Bestandteil des MRT-Befundes ◯ kein wesentlicher Bestandteil des MRT-Befundes

8 Die Erwähnung einer Invasion des Sinus cavernosus (z.B. Defekt der medialen duralen Begrenzung) ist für mich ...

Fragesupport: *Bitte wählen sie eine Antwort aus*

◯ wesentlicher Bestandteil des MRT-Befundes ◯ kein wesentlicher Bestandteil des MRT-Befundes

9 Die Klassifikation des Ausmaßes der Invasion des Sinus cavernosus mit Umwachsen des cavernösen Segmentes der Arteria carotis interna anhand der Knosp-Graduierung ist für mich ...

Fragesupport: *Bitte wählen sie eine Antwort aus*

◯ wesentlicher Bestandteil des MRT-Befundes ◯ kein wesentlicher Bestandteil des MRT-Befundes

10 Der Hinweis auf eine tumorbedingte Lumenverengung der Arteria carotis interna ist für mich ...

Fragesupport: *Bitte wählen sie eine Antwort aus*

◯ wesentlicher Bestandteil des MRT-Befundes ◯ kein wesentlicher Bestandteil des MRT-Befundes

11 Der Hinweis auf eine Erosion/Invasion der Schädelbasis (Sellaboden, Sinus sphenoidalis, Clivus) ist für mich ...

Fragesupport: *Bitte wählen sie eine Antwort aus*

- ◯ wesentlicher Bestandteil des MRT-Befundes
- ◯ kein wesentlicher Bestandteil des MRT-Befundes

12 Die Beschreibung eines reaktiven Knochenumbaus (z.B. Vergrößerung der Sella turcica, Absenkung des Sellabodens) ist für mich ...

Fragesupport: *Bitte wählen sie eine Antwort aus*

- ◯ wesentlicher Bestandteil des MRT-Befundes
- ◯ kein wesentlicher Bestandteil des MRT-Befundes

13 Die Erwähnung einer Anhebung des Diaphragma sellae ist für mich ...

Fragesupport: *Bitte wählen sie eine Antwort aus*

- ◯ wesentlicher Bestandteil des MRT-Befundes
- ◯ kein wesentlicher Bestandteil des MRT-Befundes

14 Der explizite Hinweis auf eine tumorbedingte Affektion (Verlagerung/Kompression/Ödem/Gliose) des Chiasma opticum ist für mich ...

Fragesupport: *Bitte wählen sie eine Antwort aus*

- ◯ wesentlicher Bestandteil des MRT-Befundes
- ◯ kein wesentlicher Bestandteil des MRT-Befundes

15 Eine Angabe zur Deviation des Hypophysenstiels ist für mich ...

Fragesupport: *Bitte wählen sie eine Antwort aus*

- ◯ wesentlicher Bestandteil des MRT-Befundes
- ◯ kein wesentlicher Bestandteil des MRT-Befundes

16 Angaben zur Tumorgröße sind für mich ...

Fragesupport: *Bitte wählen sie eine Antwort aus*

- ○ wesentlicher Bestandteil des MRT-Befundes in Form des maximalen Durchmessers sowie der zu dieser Achse senkrechten Ausdehnung der Raumforderung (gemessen in der Raumebene mit dem größten Tumordurchmesser)
- ○ wesentlicher Bestandteil des MRT-Befundes in Form des jeweiligen maximalen Durchmessers der Raumforderung in allen 3 Standardraumebenen (transversal, coronar, sagittal)
- ○ wesentlicher Bestandteil des MRT-Befundes in Form einer Tumorvolumetrie
- ○ wesentlicher Bestandteil des MRT-Befundes, wobei eine Klassifikation in Pikoadenom (<3 mm Durchmesser), Mikroadenom (<10 mm), Makroadenom (>10 mm) und Riesenadenom (>40 mm) ausreichend ist
- ○ kein wesentlicher Bestandteil des MRT-Befundes

17 Die Integration der Hardy-Klassifikation (Adenomgröße & Invasivität) ist für mich ...

Fragesupport: *Bitte wählen sie eine Antwort aus*

- ○ wesentlicher Bestandteil des MRT-Befundes
- ○ kein wesentlicher Bestandteil des MRT-Befundes

18 Die Beschreibung des Ausmaßes der Tumorvolumenreduktion (bei Folgeuntersuchungen nach Operation oder Einleitung einer medikamentösen Therapie) ist für mich ...

Fragesupport: *Bitte wählen sie eine Antwort aus*

- ○ wesentlicher Bestandteil des MRT-Befundes
- ○ kein wesentlicher Bestandteil des MRT-Befundes

19 Informationen zur Abgrenzbarkeit der Raumforderung (scharf/unscharf) sind für mich ...

Fragesupport: *Bitte wählen sie eine Antwort aus*

- ○ wesentlicher Bestandteil des MRT-Befundes
- ○ kein wesentlicher Bestandteil des MRT-Befundes

20 Das native T1-Signalverhalten der Läsion (in Relation zum gesunden Hypophysenvorderlappen oder zur weißen Substanz des Temporallappens ist für mich ...

Fragesupport: *Bitte wählen sie eine Antwort aus*

- ○ wesentlicher Bestandteil des MRT-Befundes
- ○ kein wesentlicher Bestandteil des MRT-Befundes bzw. mir ist die Bedeutung dieses Parameters unklar

21 Das native T2-Signalverhalten der Läsion in Relation zum gesunden Hypophysenvorderlappen oder zur weißen Substanz des Temporallappens ist für mich ...

Fragesupport: *Bitte wählen sie eine Antwort aus*

- ◯ wesentlicher Bestandteil des MRT-Befundes
- ◯ kein wesentlicher Bestandteil des MRT-Befundes bzw. mir ist die Bedeutung dieses Parameters unklar

22 Das Muster (homogen/heterogen) und die Intensität der Kontrastmittelanreicherung des Tumors in Relation zum gesunden Hypophysenvorderlappengewebe ist für mich ...

Fragesupport: *Bitte wählen sie eine Antwort aus*

- ◯ wesentlicher Bestandteil des MRT-Befundes
- ◯ kein wesentlicher Bestandteil des MRT-Befundes bzw. mir ist die Bedeutung dieses Parameters unklar

23 Das Perfusionssignal der Raumforderung ist für mich ...

Fragesupport: *Bitte wählen sie eine Antwort aus*

- ◯ wesentlicher Bestandteil des MRT-Befundes
- ◯ kein wesentlicher Bestandteil des MRT-Befundes bzw. mir ist die Bedeutung dieses Parameters unklar

24 Das Diffusionssignalverhalten der Raumforderung (ADC-Karte, apparent diffusion coefficient) ist für mich ...

Fragesupport: *Bitte wählen sie eine Antwort aus*

- ◯ wesentlicher Bestandteil des MRT-Befundes
- ◯ kein wesentlicher Bestandteil des MRT-Befundes bzw. mir ist die Bedeutung dieses Parameters unklar

25 Die Erwähnung tumorassoziierter Zysten ist für mich ...

Fragesupport: *Bitte wählen sie eine Antwort aus*

- ◯ wesentlicher Bestandteil des MRT-Befundes
- ◯ kein wesentlicher Bestandteil des MRT-Befundes

26 Die Beschreibung intratumoraler Nekrosen ist für mich ...

Fragesupport: *Bitte wählen sie eine Antwort aus*

- ◯ wesentlicher Bestandteil des MRT-Befundes
- ◯ kein wesentlicher Bestandteil des MRT-Befundes

27 Der Hinweis auf Tumoreinblutungen ist für mich ...

Fragesupport: *Bitte wählen sie eine Antwort aus*

- ◯ wesentlicher Bestandteil des MRT-Befundes
- ◯ kein wesentlicher Bestandteil des MRT-Befundes

28 Informationen zur Tumorkalzifikation sind für mich ...

Fragesupport: *Bitte wählen sie eine Antwort aus*

- ◯ wesentlicher Bestandteil des MRT-Befundes
- ◯ kein wesentlicher Bestandteil des MRT-Befundes

29 Die Beschreibung der Binnenstruktur der Raumforderung (homogen, heterogen) ist für mich ...

Fragesupport: *Bitte wählen sie eine Antwort aus*

- ◯ wesentlicher Bestandteil des MRT-Befundes
- ◯ kein wesentlicher Bestandteil des MRT-Befundes

30 Der Hinweis auf einen tumorbedingten Hydrocephalus (z.B. bei Affektion des 3. Ventrikels) ist für mich ...

Fragesupport: *Bitte wählen sie eine Antwort aus*

- ◯ wesentlicher Bestandteil des MRT-Befundes
- ◯ kein wesentlicher Bestandteil des MRT-Befundes

31 Die Beschreibung einer reaktiven Verdickung der angrenzenden Dura mater (=dural tail) ist für mich ...

Fragesupport: *Bitte wählen sie eine Antwort aus*

- ◯ wesentlicher Bestandteil des MRT-Befundes
- ◯ kein wesentlicher Bestandteil des MRT-Befundes

32 Die detaillierte Deskription anatomischer Varianten des Nasenseptums (z.B. Deviation) und der Keilbeinhöhle (z.B. verminderte Pneumatisation) ist für mich ...

Fragesupport: *Bitte wählen sie eine Antwort aus*

- ◯ wesentlicher Bestandteil des MRT-Befundes
- ◯ kein wesentlicher Bestandteil des MRT-Befundes

33 Eine ergänzende Diffusionstensorbildgebung (Fibertracking des Nervus opticus/Chiasma opticum) ist für mich ...

Fragesupport: *Bitte wählen sie eine Antwort aus*

- ◯ wesentlicher Bestandteil des MRT-Befundes
- ◯ kein wesentlicher Bestandteil des MRT-Befundes bzw. mir ist die Bedeutung dieses Parameters unklar

34 Was sollte ihrer Meinung nach die Grundlage für die Größenbestimmung eines Tumors der Sellaregion sein?

Fragesupport: *Bitte wählen sie eine Antwort aus*

- ◯ nur der Kontrastmittel aufnehmende Anteil der Raumforderung
- ◯ alle Anteile der Raumforderung inklusive Zysten und nekrotischen Arealen
- ◯ Angaben zur Tumorgröße sind aus meiner Sicht kein wesentlicher Bestandteil des MRT-Befundes

35 Als Vergleichsgrundlage bei Follow-Up-Untersuchungen sollte folgende Voruntersuchung zugrunde gelegt werden ...

Fragesupport: *Bitte wählen sie eine Antwort aus*

- ◯ erstmalige MRT-Diagnostik zum Zeitpunkt der Diagnosestellung
- ◯ erste MRT-Diagnostik nach Einleitung der Behandlung (Operation, Medikation, Bestrahlung)
- ◯ bei seriellen MRT-Voruntersuchungen unmittelbar zurückliegende MRT-Diagnostik
- ◯ eine Befundbeschreibung ohne Bezug zu Voruntersuchungen ist ausreichend

36 Die folgende Frage richtet sich an hypophysenchirurgisch tätige Kollegen: Auf welchen MRT-Sequenz(en) beruht ihre Operationsplanung?

37 Abschließend haben sie nun die Möglichkeit, Ergänzungswünsche einzubringen, die im Rahmen der Umfrage noch nicht thematisiert wurden.
